# 浙江省289例阵发性睡眠性血红蛋白尿症患者的临床特征

**DOI:** 10.3760/cma.j.cn121090-20240127-00041

**Published:** 2024-06

**Authors:** 改香 许, 伟媚 金, 宝东 叶, 松福 江, 超 胡, 昕 黄, 炳寿 谢, 慧芳 蒋, 莉莉 陈, 荣欣 姚, 滢 陆, 琳洁 李, 瑾 张, 桂芳 欧阳, 用伟 洪, 宏伟 孔, 哲君 裘, 文纪 罗, 斌斌 褚, 慧琪 张, 惠 曾, 秀杰 周, 鹏飞 施, 莹 徐, 洁 金, 红艳 佟

**Affiliations:** 1 浙江大学医学院附属第一医院，杭州 310003 The First Affiliated Hospital of Zhejiang University, School of Medicine, Hangzhou 310003, China; 2 浙江省中医院，杭州 310003 Zhejiang Provincial Hospital of Chinese Medicine, Hangzhou 310003, China; 3 温州医科大学附属第一医院，温州 325015 The First Affiliated Hospital of Wenzhou Medical University, Wenzhou 325015, China; 4 温州市人民医院，温州 325099 Wenzhou People's Hospital, Wenzhou 325099, China; 5 浙江省立同德医院，杭州 310012 Zhejiang Provincial Tongde Hospital, Hangzhou 310012, China; 6 台州市第一人民医院，台州 318020 The First People's Hospital of Taizhou, Taizhou 318020, China; 7 温州医科大学附属第二医院，温州 325035 The Second Affiliated Hospital of Wenzhou Medical University, Wenzhou 325035, China; 8 宁波市鄞州人民医院，宁波 315100 Ningbo Yinzhou People's Hospital, Ningbo 315100, China; 9 丽水市中心医院，丽水 323020 Lishui Central Hospital, Lishui 323020, China; 10 浙江大学医学院附属邵逸夫医院，杭州 310016 Sir Run Run Shaw Hospital, Zhejiang University School of Medicine, Hangzhou 310016, China; 11 宁波市第一医院，宁波 315042 Ningbo First Hospital, Ningbo 315042, China; 12 宁波市鄞州第二医院，宁波 315199 Ningbo Yinzhou Second Hospital, Ningbo 315199, China; 13 衢州市人民医院，衢州 324002 Quzhou People's Hospital, Quzhou 324002, China; 14 解放军联勤保障部队第906医院，宁波 315100 The 906 Hospital of the PLA Joint Logistics Support Force, Ningbo 315100, China; 15 萧山区第一人民医院，杭州 311201 The First People's Hospital of Xiaoshan District, Hangzhou 311201, China; 16 宁波明州医院，宁波 319199 Ningbo Mingzhou Hospital, Ningbo 319199, China; 17 湖州市第一人民医院，湖州 313099 Huzhou First People's Hospital, Huzhou 313099, China; 18 嘉兴市第一医院，嘉兴 314001 Jiaxing First Hospital, Jiaxing 314001, China; 19 海宁市人民医院，嘉兴 314499 Haining People's Hospital, Jiaxing 314499, China; 20 杭州市第一人民医院，杭州 310003 Hangzhou First People's Hospital, Hangzhou 310003, China; 21 丽水市人民医院，丽水 323050 Lishui People's Hospital, Lishui 323050, China

**Keywords:** 阵发性睡眠性血红蛋白尿症, 乳酸脱氢酶, 血栓, PNH克隆, Paroxysmal nocturnal hemoglobinuria, Lactic dehydrogenase, Thrombus, PNH clone

## Abstract

**目的:**

对浙江省阵发性睡眠性血红蛋白尿症（PNH）患者的临床特征、治疗现状及其生存情况进行多中心回顾性分析，以提高对该疾病的认识和规范化诊治水平。

**方法:**

纳入了2005年9月至2023年5月就诊于浙江省20家医院的289例PNH患者，随访并收集数据，对其临床特点、诊治现状及生存情况进行分析。

**结果:**

289例PNH患者中男148例，女141例，中位发病年龄为45（16～87）岁，发病高峰年龄为20～49岁（57.8％）。中位血清乳酸脱氢酶（LDH）水平为1 142（604～1 925）U/L。其中经典型PNH占70.9％，合并骨髓衰竭性疾病（PNH/BMF）型占24.4％，亚临床型PNH占4.7％。病程中主要临床表现包括疲劳或无力（80.8％，235/289）、头晕（73.4％，212/289）、尿色加深（66.2％，179/272）和黄疸（46.2％，126/270）。常见的合并症为血红蛋白尿（58.7％）、肾功能损害（17.6％）、血栓（15.0％）。有82.3％患者接受糖皮质激素治疗，70.9％需要输血，30.7％使用免疫抑制剂，13.8％接受抗凝治疗，6.3％接受了异基因造血干细胞移植。10年总生存（OS）率为84.4％（95％*CI* 78.0％～91.3％）。

**结论:**

PNH患者好发于中青年，男女比例相当，临床常见表现为疲劳、头晕、血红蛋白尿、黄疸、肾功能损害、反复血栓等。本组患者的10年OS率和国内其他中心报道相当。

## Abstract (en)

**Objective:**

To further improve the understanding of paroxysmal nocturnal hemoglobinuria（PNH）, we retrospectively analyzed and summarized the clinical characteristics, treatment status, and survival status of patients with PNH in Zhejiang Province.

**Methods:**

This study included 289 patients with PNH who visited 20 hospitals in Zhejiang Province. Their clinical characteristics, comorbidity, laboratory test results, and medications were analyzed and summarized.

**Results:**

Among the 289 patients with PNH, 148 males and 141 females, with a median onset age of 45（16–87）years and a peak onset age of 20–49 years（57.8％）. The median lactic dehydrogenase（LDH）level was 1 142（604–1 925）U/L. Classified by type, 70.9％（166/234）were classical, 24.4％（57/234）were PNH/bone marrow failure（BMF）, and 4.7％（11/234）were subclinical. The main clinical manifestations included fatigue or weakness（80.8％, 235/289）, dizziness（73.4％, 212/289）, darkened urine color（66.2％, 179/272）, and jaundice（46.2％, 126/270）. Common comorbidities were hemoglobinuria（58.7％）, renal dysfunction（17.6％）, and thrombosis（15.0％）. Moreover, 82.3％ of the patients received glucocorticoid therapy, 70.9％ required blood transfusion, 30.7％ used immunosuppressive agents, 13.8％ received anticoagulant therapy, and 6.3％ received allogeneic hematopoietic stem cell transplantation. The 10-year overall survival（OS）rate was 84.4％（95％ *CI* 78.0％–91.3％）.

**Conclusion:**

Patients with PNH are more common in young and middle-aged people, with a similar incidence rate between men and women. Common clinical manifestations include fatigue, hemoglobinuria, jaundice, renal dysfunction, and recurrent thrombosis. The 10-year OS of this group is similar to reports from other centers in China.

阵发性睡眠性血红蛋白尿症（PNH）是一种后天获得性造血干细胞基因突变所致的良性克隆性疾病。其发病机制主要是造血干细胞中染色体Xp22.1上PIG-A基因突变，导致血细胞膜糖化磷脂酰肌醇（GPI）锚部分或完全合成障碍，血细胞表面锚连蛋白缺失，血细胞膜对补体异常敏感易被破坏，从而引发持续性慢性血管内溶血。PNH的临床表现常有疲劳、血红蛋白尿、骨髓造血功能衰竭、血栓形成、肾功能衰竭[1]–[4]等。尽管流式细胞术的应用提高了PNH诊断的敏感性及准确性，但PNH发病多隐匿，病情轻重不一且临床表现复杂多样，尤其是合并再生障碍性贫血（AA）[5]及骨髓增生异常综合征（MDS）[6]等骨髓衰竭性疾病（BMF）而PNH症状不典型时，容易漏诊或误诊。在补体抑制剂治疗出现之前，大部分PNH管理以支持性治疗为主。现今国内补体抑制剂已经上市，可以更有效地控制溶血及改善相关并发症。目前，PNH在中国的大样本研究相对较少，对疾病特征和临床结局的认识仍不足，尤其缺乏南方地区大样本研究数据。为了加深对PNH的认识，本研究对浙江省20家医院就诊的289例PNH患者进行了回顾性分析，旨在提高对该疾病的认识和规范化诊治水平。

## 病例与方法

一、病例

本研究是一项多中心回顾性的研究，登记了2005年9月至2023年5月来自浙江省20家医院收治的295例PNH患者，根据2013年中国PNH专家共识[1]进行诊断，剔除缺失资料较多的6例患者，最终纳入289例。其中25例早期诊断的患者采用Hams试验、蔗糖溶血试验确诊。264例患者通过流式细胞术检测外周血或者骨髓CD55/CD59阴性的中性粒细胞（CD55^−^/CD59^−^ NEU）及红细胞（CD55^−^/CD59^−^ RBC），其中30例为定性结果，234例有PNH克隆大小的定量结果。根据临床特征和PNH克隆大小，将PNH患者分为经典型、骨髓衰竭疾病相关型（PNH/BMF，包括合并AA或MDS）和亚临床型。82例患者同时采用Flaer法进行了检测。病例纳入及分类流程见图1。

**图1:**
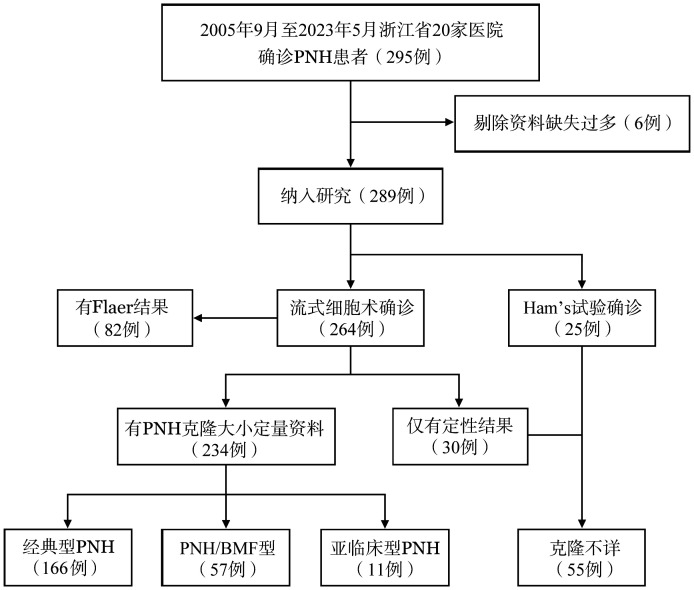
阵发性睡眠性血红蛋白尿症（PNH）患者纳入及分类流程图 **注** PNH/BMF：骨髓衰竭疾病相关型PNH

本研究收集了患者的临床表现、实验室检查、治疗、合并症和生存情况。通过流式细胞术CD55^−^/CD59^−^ NEU及CD55^−^/CD59^−^ RBC、Flaer法CD24^−^中性粒细胞及CD14^−^单核细胞检测PNH克隆大小。通过骨髓检查鉴别PNH/BMF。通过超声、CT或MRI检查血栓情况。治疗上收集了包括糖皮质激素、血制品、免疫抑制剂、抗凝治疗、异基因造血干细胞移植、化疗及补体抑制剂的使用情况。

二、随访

通过电话以及查阅患者门诊和住院病历对患者进行随访，随访截至2023年7月31日，中位随访时间为60.8（2.0～516.3）个月。总生存（OS）定义为从确诊至任何因素导致的患者死亡或者随访终点的时间。

三、统计学处理

采用SPSS 26.0软件进行数据分析。计量资料采用*M*（范围）或*M*（*Q*1,*Q*3）表示，计数资料采用例数（％）表示。对数据进行组间比较时，连续变量正态分布数据采用独立样本*t*检验，非正态分布数据采用Mann-Whitney *U*检验，分类变量进行卡方检验或Fisher精确概率法。双侧*P*<0.05为差异有统计学意义。

## 结果

一、临床特征

289例PNH患者纳入研究，其中男148例，女141例，男女比为1.05∶1。病程从2个月到44年不等。中位年龄45（16～87）岁，20～49岁为高发群体，占57.8％，年龄分布见图2。由于资料来自不同中心且是回顾性研究，一些患者病史较长，有些数据无法追溯，根据有记录的临床数据，在病程中主要临床表现包括疲劳或无力81.3％（235/289）、头晕73.4％（212/289）、尿色加深65.8％（179/272）、黄疸46.7％（126/270）、黏膜出血20.2％（34/168）、呼吸困难20.9％（54/258）、疼痛17.0％（44/259）、男性勃起功能障碍13.6％（16/118）、心悸胸闷10.2％（16/157）、吞咽困难1.5％（4/259）。51.7％（140/272）的患者发生过血红蛋白尿，发作频率自每年1～2次至每天1～2次。初诊时就诊前三位科室依次为血液科、肾内科、消化科，其次为急诊科、泌尿外科、肝病科、呼吸内科、神经内科等。

**图2:**
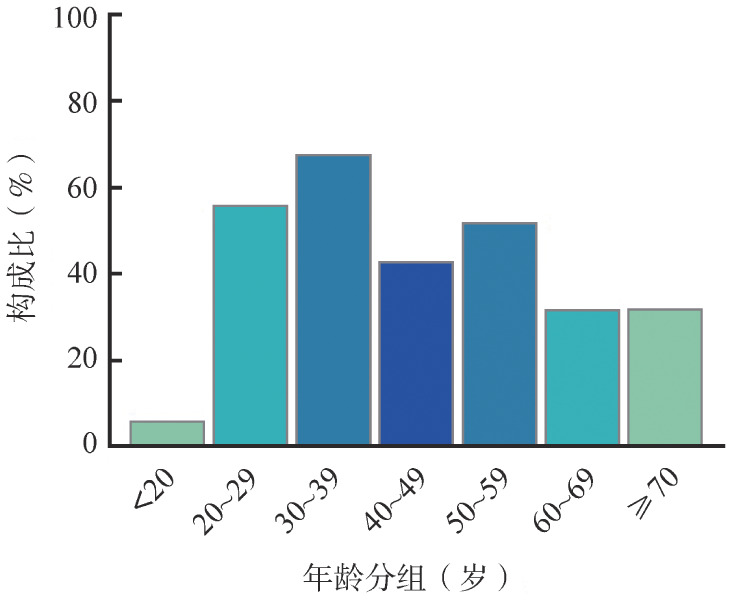
浙江省289例阵发性睡眠性血红蛋白尿症患者年龄分布

289例患者中，经典型PNH占70.9％（166例），PNH/BMF型占24.4％（57例），亚临床型PNH占4.7％（11例），另55例患者类型无法确定（表1）。对3组患者初诊时临床特征进行分析，结果显示经典型PNH患者相较于PNH/BMF型更容易发生男性勃起功能障碍（*P*＝0.013），有更高的罹患肺动脉高压比例（*P*＝0.012）及更高的血红蛋白尿比例（*P*＝0.002）（表1）。

**表1:** 浙江省289例阵发性睡眠性血红蛋白尿症（PNH）患者初诊时基线特征

基线特征	总队列（289例）	经典型PNH（166例）	PNH/BMF（57例）	亚临床型PNH（11例）	克隆不祥（55例）	*P*值^a^
HGB[g/L，*M*（*IQR*）]	67（54，82）	67（53，81）	67（51，80）	67（57，81）	63（46，116）	0.345
PLT[×10^9^/L，*M*（*IQR*）]	80（40，150）	83（49，153）	86（35，152）	42（32，104）	78（52，145）	0.023
WBC[×10^9^/L，*M*（*IQR*）]	3.5（2.5，5.3）	4.1（2.9，5.3）	2.8（2.3，4.2）	3.8（2.8，5.2）	3.7（2.5，4.9）	0.780
乳酸脱氢酶^b^ [U/L，*M*（*IQR*）]	1142（604，1925）	1202（744，1804）	1074（670，2014）	391（520，1434）	1342（488，1991）	0.015
粒细胞 CD59^− c^[%，*M*（范围）]	44.7（1.2~100.0）	69.7（2.1~100.0）	13.0（3.2~76.9）	1.4（1.0~9.0）	–	0.012
红细胞 CD59^− c^[%，*M*（范围）]	45.2（0.8~99.9）	56.9（0.9~99.9）	20.7（1.3~49.7）	1.6（3.5~10.0）	–	0.014
粒细胞 CD55^− c^[%，*M*（范围）]	35.1（0.3~99.7）	53.3（0.8~99.7）	25.3（2.5~48.0）	1.7（1.1~8.0）	–	0.037
红细胞 CD55^− c^[%，*M*（范围）]	44.3（1.9~99.5）	58.9（3.9~99.5）	21.7（3.2~49.5）	1.2（0.5~6.7）	–	0.021
Flaer^−^/CD14^− d^[%，*M*（范围）]	91.3（0.7~99.4）	85.3（0.7~99.4）	21.1（7.1~32.5）	–	–	0.011
Flaer^−^/CD24^− d^[%，*M*（范围）]	84.9（0.1~99.8）	91.3（1.2~99.8）	20.7（0.1~30.5）	–	–	0.014
腹痛史[阳性例数/总例数（%）]	23/221（10.4）	17/155（11.1）	4/45（8.9）	1/12（8.4）	1/9（11.1）	0.022
吞咽困难病史[阳性例数/总例数（%）]	4/254（1.6）	2/157（1.3）	1/50（2.0）	0（0）	1/47（2.1）	0.055
呼吸急促史[阳性例数/总例数（%）]	53/246（21.5）	29/160（18.1）	15/48（31.2）	1/11（9.1）	8/27（29.6）	0.023
勃起功能障碍史[阳性例数/总例数（%）]	16/114（14.0）	12/67（17.9）	3/27（11.1）	0（0）	1/20（5.0）	0.013
疲劳史[阳性例数/总例数（%）]	211/261（80.8）	125/163（76.7）	47/55（85.4）	6/8（75.0）	33/35（94.3）	0.005
肺动脉高压病史[阳性例数/总例数（%）]	20/51（39.2）	15/33（45.4）	4/12（33.3）	0（0）	1/6（16.7）	0.012
血红蛋白尿病史[阳性例数/总例数（%）]	81/138（58.7）	49/70（70.4）	19/34（55.9）	2/7（28.6）	11/27（40.7）	0.002

**注** PNH/BMF：骨髓衰竭疾病相关型PNH；–：无数据。a：统计学分析仅比较经典型PNH、PNH/BMF及亚临床型PNH；b：251例患者有该结果，其中经典型PNH、PNH/BMF、亚临床型PNH及克隆不详分别有166、40、11、34例；c：234例患者有该结果，其中经典型PNH、PNH/BMF、亚临床型PNH分别有166、57、11例；d：82例患者有该结果，其中经典型PNH、PNH/BMF、亚临床型PNH分别有76、6、0例

二、实验室检查结果

血常规结果显示中，中位HGB 67（54，82）g/L、PLT 80（40，150）×10^9^/L、WBC 3.5（2.5，5.3）×10^9^/L。所有患者均有血象异常，表现为单系或多系血细胞减低，其中44.6％（129/289）的患者表现为三系减少，10.4％（30/289）表现为红系和粒系同时减少，1.0％（3/289）表现为粒系和血小板同时减少。中位乳酸脱氢酶（LDH）1142（604，1925）U/L。245例患者有尿常规记录，129例（52.65％）尿隐血阳性。在234例有PNH克隆定量数据患者中，PNH克隆大小分别为：CD59^−^ RBC 45.2％（0.8％～99.9％），CD55^−^ RBC 44.3％（1.9％～99.5％），CD59^−^ NEU 44.7％（1.2％～100.0％），CD55^−^ NEU 35.1％（0.3％～99.7％）。244例患者进行了骨髓检查，其中146例（59.8％）骨髓增生活跃或极度活跃，98例（40.2％）骨髓增生减低或极度减低。合并AA和MDS比例分别为40.2％（98/244）和9.4％（23/244）。

三、合并症

239例有肾功能结果患者中，42例（17.6％）合并肾功能损害，其中8例为肾功能失代偿期，5例为肾衰竭期，2例为氮质血症期，1例为尿毒症期，26例（10.9％）患者缺乏肾功能不全定量数据。

206例患者追溯了血栓发生情况，有31例（15.0％）合并血栓，其中9例（29.0％）为脑血栓形成，8例（25.8％）为下肢深静脉血栓形成，6例（19.4％）为腹部血栓形成，4例（12.9％）为肺栓塞形成，1例（3.2％）为心肌梗死，3例（9.7％）为其他部位血栓形成。有2例（6.4％）患者同时出现多部位血栓形成（脑血栓及双下肢深静脉血栓形成）。在已明确血栓部位的患者中，动脉与静脉血栓发生比例为1∶1（14∶14）。

168例追溯了出血发生情况，有34例（20.2％）发生了出血事件，其中12例为皮肤黏膜出血，8例为女性月经过多，7例为消化道出血，7例为球结膜或者眼底出血。在可追溯的患者中，发生男性勃起功能障碍14.0％（16/114），胆囊炎13.5％（34/252）。

四、治疗

254例患者收集到了详细治疗情况，209例（82.3％）接受过糖皮质激素治疗；180例（70.9％）接受过输血治疗；78例（30.7％）接受过免疫抑制剂治疗，治疗药物包括环孢素A、他克莫司及环磷酰胺；35例（13.8％）接受过抗凝治疗，治疗药物包括低分子肝素及利伐沙班，其中4例预防性使用利伐沙班抗凝；16例（6.3％）接受了异基因造血干细胞移植；6例（2.4％）接受过化疗；22例（8.6％）使用了补体抑制剂，其中19例参加了C5补体抑制剂的临床试验，3例使用了上市的依库珠单抗。

五、生存分析

本研究共纳入289例患者，其中43例生存情况不详，因此对246例有生存数据的患者进行了生存分析。中位随访时间为60.8（2.0～516.3）个月，5年OS率为91.3％（95％*CI* 87.2％～95.5％），10年OS率为84.4％（95％*CI* 78.0％～91.3％）（图3A）。随访期内，共有28例患者死亡，死亡患者的中位年龄60（26～88）岁，其中年龄>49岁患者占71.4％（20/28）。死亡患者中16例为经典型，2例为PNH/BMF型，10例为克隆不详。死亡原因包括心脑血管意外（出血或者栓塞）8例、重症肺炎3例、多脏器功能衰竭3例、移植相关死亡1例、肾衰竭1例、二次肿瘤后死亡1例，另11例患者死因不详。伴有血栓形成的31例患者10年OS率为78.7％（95％*CI* 60.9％～99.9％），明确无血栓的175例患者10年OS率85.4％（95％*CI* 77.9％～93.8％）（*P*＝0.052）（图3B）。伴有肾功能损害42例患者，数据收集时2例患者生存情况不详，余40例10年OS率为71.3％（95％*CI* 56.2％～90.3％），174例无肾功能损害患者10年OS率85.9％（95％*CI* 77.9％～94.7％）（*P*＝0.070）（图3C）。

**图3:**
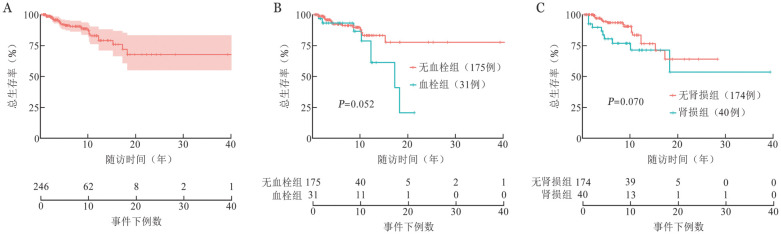
浙江省阵发性睡眠性血红蛋白尿症患者生存分析 **A** 246例有生存数据患者的总生存情况；**B** 明确有血栓形成和明确无血栓形成患者生存分析比较；**C** 明确有肾功能损害和明确无肾功能损害患者生存分析比较

## 讨论

PNH是后天获得的一种良性克隆性血管内溶血性疾病，表现为反复溶血、骨髓衰竭、血栓形成、肾功能不全等，影响患者的生活质量及生存。早期文献报道显示，PNH患者的10年OS率仅为50％～65％[7]–[8]，尽管随着对该疾病的认识及诊断水平的提高，患者的生存得到一定程度的改善，但是在使用补体抑制剂之前，患者的生存仍较差。由于大部分是回顾研究，报道的OS率存在一定差异。Fu等[9]报道我国北方单中心92例PNH患者的10年OS率为70.77％[9]，和国外报道相近[10]–[11]。但Du等[12]报道的来自我国北方另一家中心512例患者，经典型和PNH/BMF型的10年OS率分别为93.2％和83.7％。本组数据显示，10年OS率为84.4％，由于这是一项多中心的回顾性研究，跨越时间比较长，一部分早期患病且死亡患者可能未进行登记，长生存分析会有较强的选择偏倚，我们认为真实的10年OS率应该低于统计结果。

本研究分析了以浙江省为代表的南方地区289例PNH患者的临床特征、治疗和疾病转归。结果显示，男女发病比例为1.05∶1，中位发病年龄为45岁，发病年龄高峰为20～49岁，在男女比例和发病年龄上和国内外报道一致[7],[9],[12]–[13]。本研究显示，PNH患者的临床表现复杂且多样，但均有不同程度的贫血症状，主要表现为乏力、头晕、气促等不适。且患者有较高的血管内溶血发生率，51.7％的患者在病程中的任何时间点有肉眼可见的血红蛋白尿，以经典型为主。

血栓栓塞是PNH患者最常见的死亡原因，占已知死亡原因的40％～67％[7],[13]–[15],[18]。国外报道显示28％～44％的PNH患者在其病程中至少发生过1次血栓栓塞事件[7]–[8],[10],[15]–[17],[19]–[20]，这一比例高于中国人群的13.6％～18.3％[9],[12]–[13]。PNH患者血栓形成机制涉及多个方面，包括血小板活化、游离血红蛋白毒性、一氧化氮耗竭、缺乏其他糖基磷脂酰肌醇连接蛋白（如尿激酶型纤溶酶原激活剂受体）和内皮功能障碍等[14],[21]–[24]。本组数据显示，发生血栓事件PNH患者占15.0％（31/206），在已知死因的17例患者中，有7例（41.2％）合并血栓栓塞事件。本组患者血栓发生率和国内[9],[12]–[13]及亚洲其他国家[17]–[18]报道数据相近。亚洲人群血栓发生率低于西方国家的原因尚不清楚，可能与种族特征相关。此外，国内PNH诊断率较低和误诊率较高，对并发症的筛查力度不够，以及出现血栓为首发症状的患者可能就诊于其他科室等综合原因可能会影响血栓的实际发生率。本研究我们发现，31例明确有血栓事件的患者10年OS率为78.7％，而175例明确无血栓形成的患者10年OS率为85.4％（*P*＝0.052）。本组患者血栓常见部位包括脑静脉、下肢深静脉和腹部静脉，与文献报道相似[12]–[13],[16]–[17]。虽然Hall等[25]报道，在血栓高风险的PNH患者中进行预防性抗凝治疗可以降低血栓形成的风险，但长期抗凝治疗存在一定的致命性出血风险。患者是否需要以及如何进行预防性抗凝治疗尚需进一步探索，目前尚未达成一致的专家共识。C5补体抑制剂可减少血栓形成的风险，既往研究报道，通过C5补体抑制剂依库珠单抗的治疗，显著降低了血栓事件的发生风险及死亡风险[14]。

肾功能损害是PNH患者另一常见并发症，从机制上也是多因素共同作用的结果。溶血所产生的血浆游离血红蛋白或微血栓可能反复作用于肾组织，导致一氧化氮的消耗，进而增加肾动脉阻力，加上感染和肾损伤性药物的使用等因素的影响[26]。既往报道显示20％～30％的PNH患者会出现不同程度的肾脏损害，表现为蛋白尿、血尿、肌酐升高或肌酐清除率下降[4],[17],[27]。肾功能衰竭被认为是8％～18％的人群死亡的主要原因[17]。在本研究中，我们观察到17.6％的患者合并肾功能损害。在已知死因的17患者中，有3例死于包括肾功能衰竭在内的多脏器功能损害，1例死于单纯肾衰竭，提示肾功能衰竭患者具有较高的死亡风险。本研究我们发现，40例伴有肾功能损害的患者10年OS率为71.3％（95％*CI* 56.2％～90.3％），174例无肾功能损害患者10年OS率85.9％（95％*CI* 77.9％～94.7％）（*P*＝0.070）。

本组患者中，大部分接受了对症支持治疗，82.3％的患者接受糖皮质激素治疗，高于国内其他中心的报道（30.9％）[12]。在补体抑制剂上市之前，糖皮质激素是PNH患者急性溶血时的主要治疗药物。依据中国专家共识[1]及国内文献报道[9]，接受糖皮质激素治疗的患者在1年内的总有效率为79.1％。但是糖皮质激素长期使用给患者带来的不良反应需要引起足够的重视。此外，本组患者接受输血及免疫抑制剂治疗的比例与国内其他中心相比也相对较高，推测与本组患者接受更积极的治疗有关。

总之，这是国内首次大样本报道南方区域性PNH患者流行病学的数据，由于是回顾性研究，数据收集不完整，对客观结果存在影响，但仍为临床提供了关于南方PNH患者发病特征、治疗现状及生存的情况。浙江省已经成立了PNH协作组，我们期待通过协作组的努力，进一步实现对浙江省PNH患者的早期诊断、合理治疗及规范管理，这将有助于提高患者的生活质量及长期生存率。随着补体抑制剂在中国的上市，将极大改善PNH患者的生存状况。早期识别、及时诊断和规范性治疗对PNH患者的管理尤为重要。
